# Spinal pathways involved in somatosensory inhibition of the psychomotor actions of cocaine

**DOI:** 10.1038/s41598-017-05681-7

**Published:** 2017-07-13

**Authors:** Suchan Chang, Yeonhee Ryu, Young Seob Gwak, Nam Jun Kim, Jin Mook Kim, Jun Yeon Lee, Seol Ah Kim, Bong Hyo Lee, Scott C. Steffensen, Eun Young Jang, Chae Ha Yang, Hee Young Kim

**Affiliations:** 1 College of Korean Medicine, Daegu Haany University, Daegu, 42158 South Korea; 2 Korean Medicine Fundamental Research Division, Korea Institute of Oriental Medicine, Daejeon, 34054 South Korea; 3 Department of Psychology and Neuroscience (1050 SWKT), Brigham Young University, Provo, UT 84602 USA

## Abstract

Previous studies have demonstrated that somatosensory stimuli influence dopamine transmission in the mesolimbic reward system and can reduce drug-induced motor behaviors, craving and dependence. Until now, the central links between somatosensory and brain reward systems are not known. Here, we show that the dorsal column (DC) somatosensory pathway contains projections that convey an inhibitory input from the periphery to mesolimbic reward circuits. Stimulation of the ulnar nerve under HT7 acupoint suppressed psychomotor response to cocaine, which was abolished by disruption of the DC pathway, but not the spinothalamic tract (STT). Low-threshold or wide-dynamic range neurons in the cuneate nucleus (CN) were excited by peripheral stimulation. Lesions of dorsal column or lateral habenula (LHb) prevented the inhibitory effects of peripheral stimulation on cocaine-induced neuronal activation in the nucleus accumbens (NAc). LHb neurons projecting to the ventral tegmental area (VTA)/rostromedial tegmental nucleus (RMTg) regions were activated by peripheral stimulation and LHb lesions reversed the inhibitory effects on cocaine locomotion produced by peripheral stimulation. These findings suggest that there exists a pathway in spinal cord that ascends from periphery to mesolimbic reward circuits (spino-mesolimbic pathway) and the activation of somatosensory input transmitted via the DC pathway can inhibit the psychomotor response to cocaine.

## Introduction

Much research has sought to elucidate the neurobiological mechanisms underlying reward and addiction. The majority of this work has focused on the traditional mesolimbic reward circuitry in the brain. This pathway originates with dopamine (DA) neurons in the ventral tegmental area (VTA) and projects primarily to the nucleus accumbens (NAc) in the ventral striatum, but it also projects to the amygdala, the bed nucleus of stria terminalis, the lateral septal area and the lateral hypothalamus^1^.

The activity of VTA DA neurons is inhibited or excited by somatosensory stimuli, such as tactile or noxious stimulation^2, 3^. Tactile stimulation of the forelimb inhibits DA release in the contralateral striatum^4, 5^, while noxious stimulation applied to the tail enhances DA release in the NAc^6^, suggesting that the mesolimbic reward system is modulated by somatosensory input. We and others have shown that sensory stimulation reduces drug craving behaviors through the modulation of the mesolimbic DA systems in rats and humans^7–9^. The application of acupuncture, widely accepted as a form of peripheral sensory stimulation^10^, to the ulnar tunnel (also called as HT7 *Shenmen Acupoint* or Guyon’s Canal) decreases DA release in the NAc by activating GABA neurons in the VTA, resulting in the suppression of cocaine, morphine and ethanol self-administration^11–13^. We have previously reported that inhibition of drug-seeking behaviors is mediated via activation of specific fiber types in the ulnar nerve. Mechanical stimulation of the ulnar nerve activates peripheral sensory afferents, such as Pacinian and Meissner corpuscles, which are conveyed via large A-fibers within the nerve trunk, and inhibits the locomotor activating effects of cocaine^11^, providing further evidence that the activation of primary sensory fibers can attenuate the reinforcing effects of drugs of abuse in the reward system. However, the central links between somatosensory and brain reward systems are not completely understood.

There are two main somatosensory pathways in the spinal cord that relay sensory signals from the periphery to brain. The dorsal column (DC)-medial lemniscus pathway transmits innocuous tactile information, while the spinothalamic tract (STT) conveys noxious signals, including pain and temperature^14^. As there is compelling evidence that innocuous or noxious stimulation influences the mesolimbic DA system^2, 15^, the spinal ascending pathways, such as DC and STT, likely play a role in linking the peripheral sensory system to reward circuits. In addition, the lateral habenula (LHb), a key epithalamic structure interconnecting sensory inputs to mesolimbic DA systems, has been reported to be critically involved in processing of peripheral sensory inputs and reward^16–18^.

To identify the ascending spinal pathway contributing to peripheral stimulation-induced inhibition of drug-induced behaviors, we hypothesized that: 1) Selective lesions of the DC or STT pathways would affect sensory stimulation-induced inhibition of cocaine locomotor activity; 2) DC nuclei would be activated by specific peripheral stimulation; 3) Surgical transection of the DC or STT pathways would decrease the inhibition of cocaine modulation of the NAc by peripheral stimulation; 4) Peripheral stimulation would activate the lateral habenula (LHb); and 5) LHb lesions would disrupt sensory stimulation-induced effects on cocaine locomotion or neuronal activation in NAc.

## Results

### Mechanical Stimulation of the Ulnar Nerve Suppresses Cocaine-induced locomotor Activity

**Figure 1:**
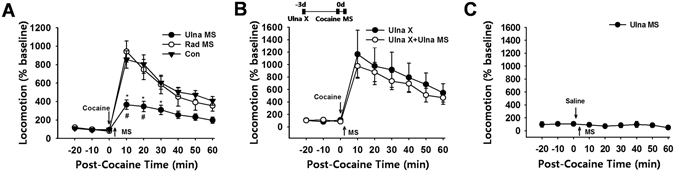
Mechanical stimulation of the ulnar nerve suppresses cocaine-induced locomotor activity. (**A**) Mechanical stimulation (MS) of the ulnar site (Ulna MS; *n* = 6) significantly attenuated the locomotor response to cocaine, compared to the control group that received cocaine only (Con; *n* = 6) *p < 0.05 vs. Con; ^#^ *p* < 0.05 vs. Rad MS. Mechanical stimulation of the radial site (Rad MS; *n* = 6) did not alter cocaine-induced locomotion. (**B**) The inhibitory effect of ulnar MS on cocaine locomotion was not observed in rats with ulnar nerve injury (Ulna X + Ulna MS; *n* = 8) when compared to control rats with ulnar nerve injury (Ulna X; *n* = 8). The term ‘X’ denotes lesion or injury. (**C**) Ulna MS did not affect locomotion in the control rats injected with saline (Ulna MS; n = 5).

Systemic injection of cocaine (15 mg/kg, i.p.) markedly increased locomotor activity, which lasted for approximately 60 min from the peak magnitude at 10 min (Con; Fig. 1A). Mechanical stimulation (MS) of the ulnar area (Ulna MS) near HT7 acupoint for 20 sec, which is sufficient to activate A- and C-fibers^11^, attenuated cocaine enhancement of locomotor activity, while MS of the radial site did not alter cocaine-induced locomotor activity (Rad MS; Fig. 1A). Mechanical stimulation of the ulnar site after sectioning the ulnar nerve had no effect on cocaine-induced locomotion (Fig. 1B; *p* > 0.05), suggesting that somatosensory-induced inhibition of cocaine-induced locomotion was mediated through activation of the ulnar nerve. Ulna MS did not affect locomotor activity in saline-injected rats (Fig. 1C), suggesting that Ulna MS attenuated cocaine-evoked locomotion, but not generalized locomotion. As ulnar MS produced consistent and reproducible inhibitory effects on cocaine-induced locomotion in our previous^11^ and present studies; MS was applied only to the ulnar site in subsequent experiments.

### Dorsal Column (DC) Pathways Mediate Ulnar Nerve Stimulation-Induced Inhibition of Cocaine Locomotion

**Figure 2:**
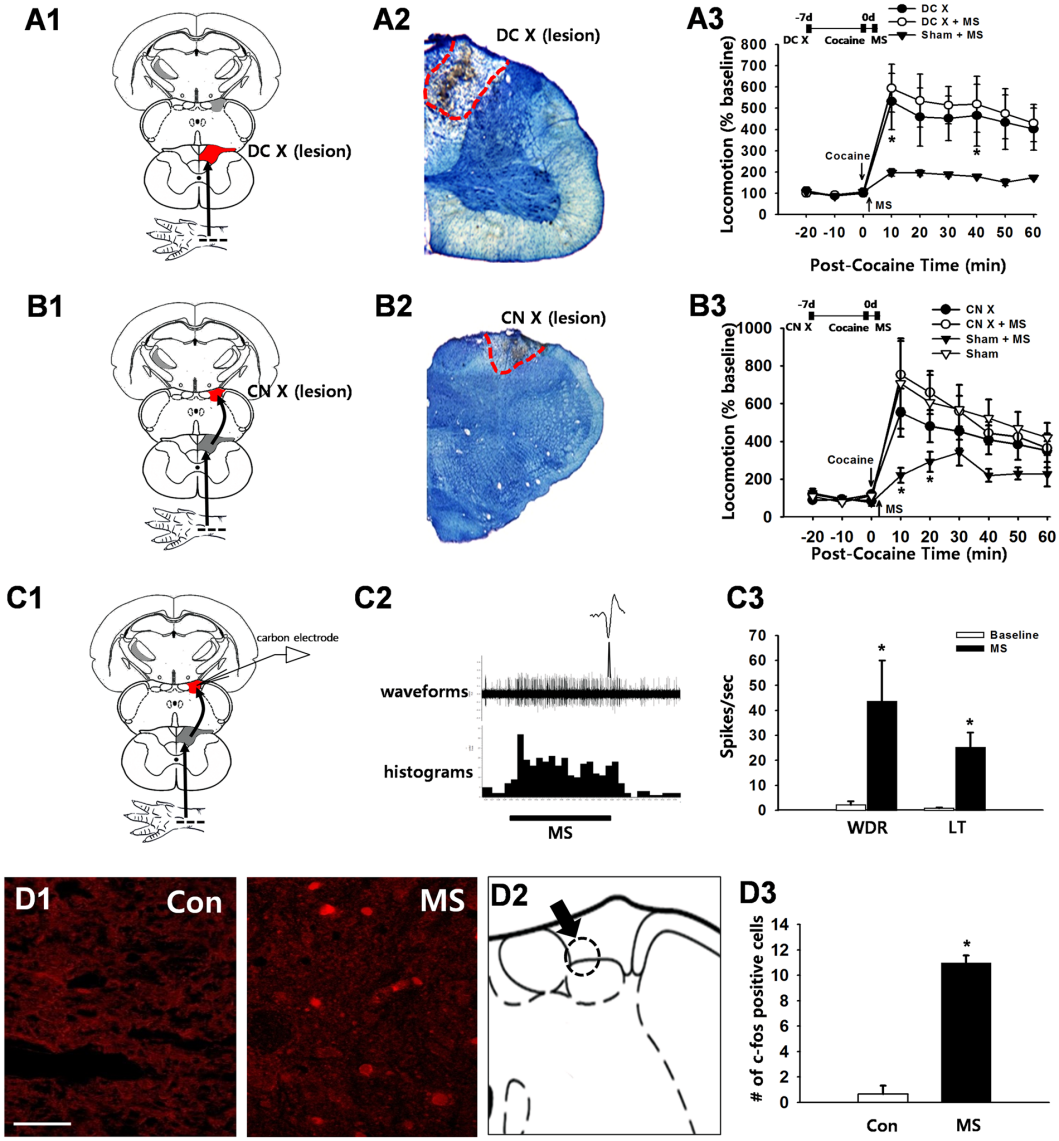
The role of the dorsal column somatosensory pathway in ulnar nerve inhibition of cocaine-induced locomotion. (**A**) Dorsal column lesions (DC X) were introduced bilaterally at the C3 level of the spinal cord (A1), as shown in a representative image of toluidine blue staining (A2). Mechanical stimulation (MS) of the ulnar site (MS) reduced cocaine-induced locomotion (Sham + MS; *n* = 10), compared to the control group (DC X; *n* = 6), which was blocked by surgical lesion of DC prior to MS (DC X + MS; n = 7; A3). *p < 0.05 vs. Sham + MS. (**B**) When CN lesions (CN X) were performed bilaterally 7–10 days prior to the experiment (B1 and B2), MS of the ulnar site failed to reduce cocaine locomotion (CN X + MS; *n* = 10), compared to the control groups of CN X (CN X; *n* = 5), Sham (*n* = 5) and Sham + MS (*n* = 5; B3). *p < 0.05 vs. CN X + MS. (**C**) Representative waveforms and peri-stimulus time histograms of *in vivo* extracellular recordings in CN neurons (C1–2). Ulnar MS markedly increased CN WDR (*n* = 6) and LT (*n* = 7) spiking activity (C3). *p < 0.05 vs. Baseline. (**D**) Increased expression of c-fos immunopositive cells following ulnar stimulation (MS, *n = *6; D1 & D3) in the dorsomedial portion of the CN (D2), compared to the control group (Con, *n* = 6; D1). Bar = 20 µm. *p < 0.05 vs. Con (D3).

To evaluate the role of the DC somatosensory pathway in ulnar MS-induced inhibition of cocaine locomotion, bilateral lesions of the DC were made at the C3 level 7–10 days before testing (Fig. 2A1 and A2). Inhibition of cocaine locomotion by ulnar MS was prevented by DC lesions (the term ‘X’ refers to ‘lesion’; two-way ANOVA: group factor *F* = 4.095, *p* = 0.050; time factor *F* = 6.202, *p* < 0.001; interaction *F* = 0.603, *p* = 0.804; Fig. 2A3), or by bilateral lesions of the CN, the second-order neurons in the DC pathway (*p* < 0.05, CN X + MS vs. Sham + MS, Fig. 2B1–3). Taken together, these results indicate that the DC pathway mediates ulnar MS-induced inhibition of cocaine locomotion.

**Figure 3:**
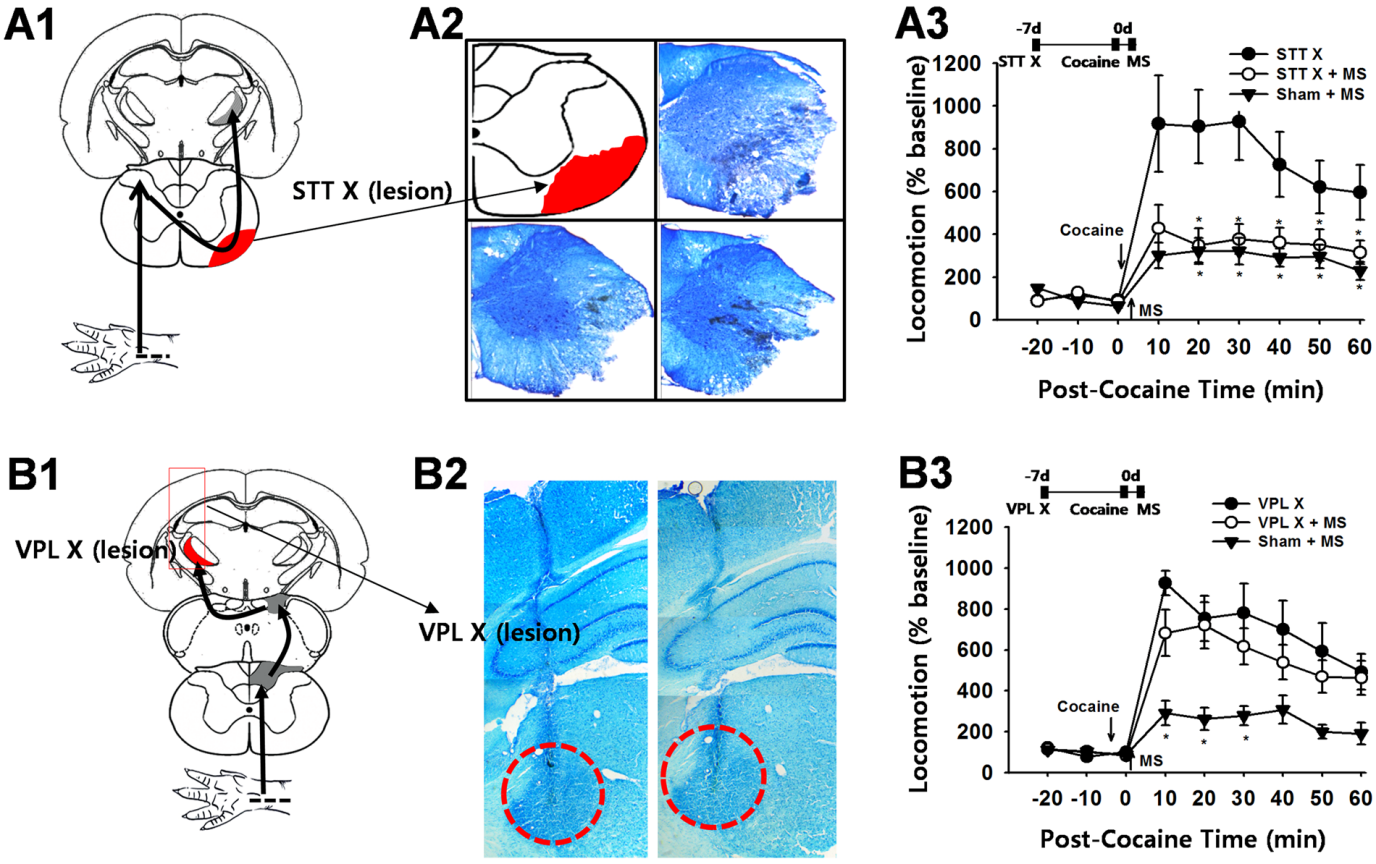
The effects of STT or VPL lesions on the ulnar inhibition of cocaine locomotion. (**A**) STT lesions (STT X) at the C3 level of the spinal cord (A1–2) did not affect ulnar MS-induced inhibition of cocaine-induced locomotion (STT X + MS; *n* = 8) when compared to the control group (STT X; *n* = 7) or ulnar stimulation without STT lesion (Sham + MS; *n* = 6; A3). *p < 0.05 vs. STT X. Representative images of STT lesions stained by toluidine blue (A2). (**B**) Bilateral chemical lesions of the VPL (B1–2) block the inhibitory effects of cocaine locomotion by ulnar MS (VPL X + MS; *n = *7), compared to the control groups of VPL X (*n* = 7) and Sham + MS (*n* = 6; B3). *p < 0.05 vs. Sham + MS or VPL X + MS.

To determine if ulnar MS activates the CN directly, we performed single-unit extracellular recordings in anesthetized rats (Fig. 2C). Figure 2C2 shows the waveforms and the peri-stimulus spike histogram during the recording of a CN neuron. Of 13 recorded CN neurons, 6 were classified as having a wide dynamic range (WDR), 7 neurons were characterized as having a low threshold (LT), and 0 high threshold (HT) neurons were found. The baseline activity of LT and WDR neurons were 0.8 ± 0.3 spikes/sec and 2.1 ± 1.5 spikes/sec, respectively. Ulnar MS markedly increased the firing rate of CN LT neurons to 25.2 ± 5.98 Hz and WDR neurons to 43.6 ± 16.4 Hz (*p* < 0.05, Fig. 2C3). To identify the region of the CN activated by ulnar MS, we examined c-fos expression in the CN after stimulation in another subset of rats. The number of c-fos immunopositive cells was significantly increased in the CN after ulnar MS (11 ± 0.57) compared to control groups (Con, 0.66 ± 0.66) (Fig. 2D1–3, *p* < 0.05) and were detected mainly in the dorsomedial portion of the CN (Fig. 2D2).

### The Spinothalamic Tract (STT) Pathway is not Involved in the Inhibitory Effects of Ulnar Nerve Stimulation on Cocaine Responses

To evaluate the role of the STT pathway in the inhibition of cocaine-induced locomotor activity by ulnar MS, STT lesions were made by surgically cutting the ventrolateral columns at the C3 level 7–10 days before testing (Fig. 3A1 and A2). STT lesions did not alter the inhibitory effects of ulnar MS on cocaine locomotion (*p* > 0.05, STT X + MS vs. Sham + MS, Fig. 3A3), suggesting that the STT pathway does not play a role in these processes (Fig. 3A). In an experiment to evaluate the role of the VPL, the third-order neurons of the DC pathway, in the inhibition of cocaine-induced locomotor activation by ulnar MS, chemical lesion of the VPL by ibotenic acid (Fig. 3B1 and B2) effectively blocked the ulnar MS effects (VPL X + MS vs. Sham + MS; two-way ANOVA: group factor *F* = 7.010, *p* = 0.017; time factor *F* = 14.122, *p* < 0.001; interaction *F* = 2.584, *p* = 0.016; Fig. 3B3), confirming the VPL mediation of DC input.

### Lateral Habenula (LHb) Projections to the VTA/RMTg Mediate the Inhibitory Effects of Ulnar Nerve Stimulation on Cocaine Responses

**Figure 4:**
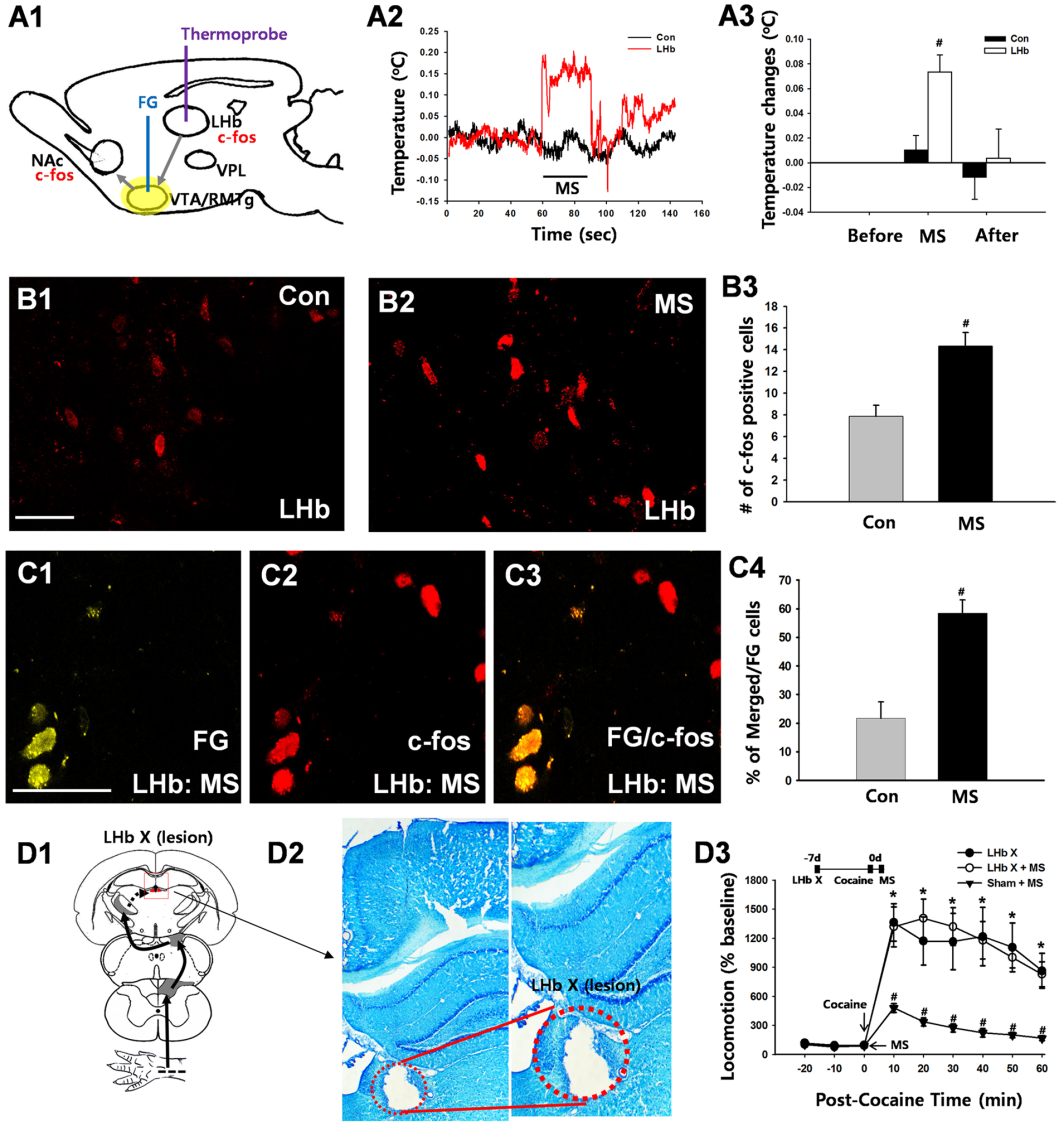
The role of VTA/RMTg projections to the lateral habenula (LHb) on ulnar inhibition of cocaine locomotion. (**A**) Ulnar MS increases LHb temperature (A1–3). (**B**) Activation of LHb neurons projecting to the VTA/RMTg regions following ulnar stimulation. The number of c-fos positive cells in the LHb increased by approximately 2-fold in ulnar MS rats (MS, B2-B3; *n* = 5) compared to rats in the control group (Con; B1; *n* = 5). (**C**) c-fos LHb positive cells (C2) were labeled retrogradely with fluorogold (FG; yellow; C1) injected into the VTA/RMTg regions (C1–3). The number of c-fos positive cells double-labeled with FG significantly increased in ulnar MS (n = 5), compared to control (Con; n = 5; C4). ^#^ *p* < 0.05 vs. Con. (**D**) Electrolytic lesions of LHb (LHb X; D1–2) prevented ulnar MS-induced inhibition of cocaine-induced locomotion (LHb X + MS; *n* = 5), compared to the control groups of LHb lesion only (*n* = 5) and Sham + MS (*n* = 5) (D3). ^#^ *p* < 0.05 vs. LHb X. *p < 0.05 vs. Sham + MS.

**Figure 5:**
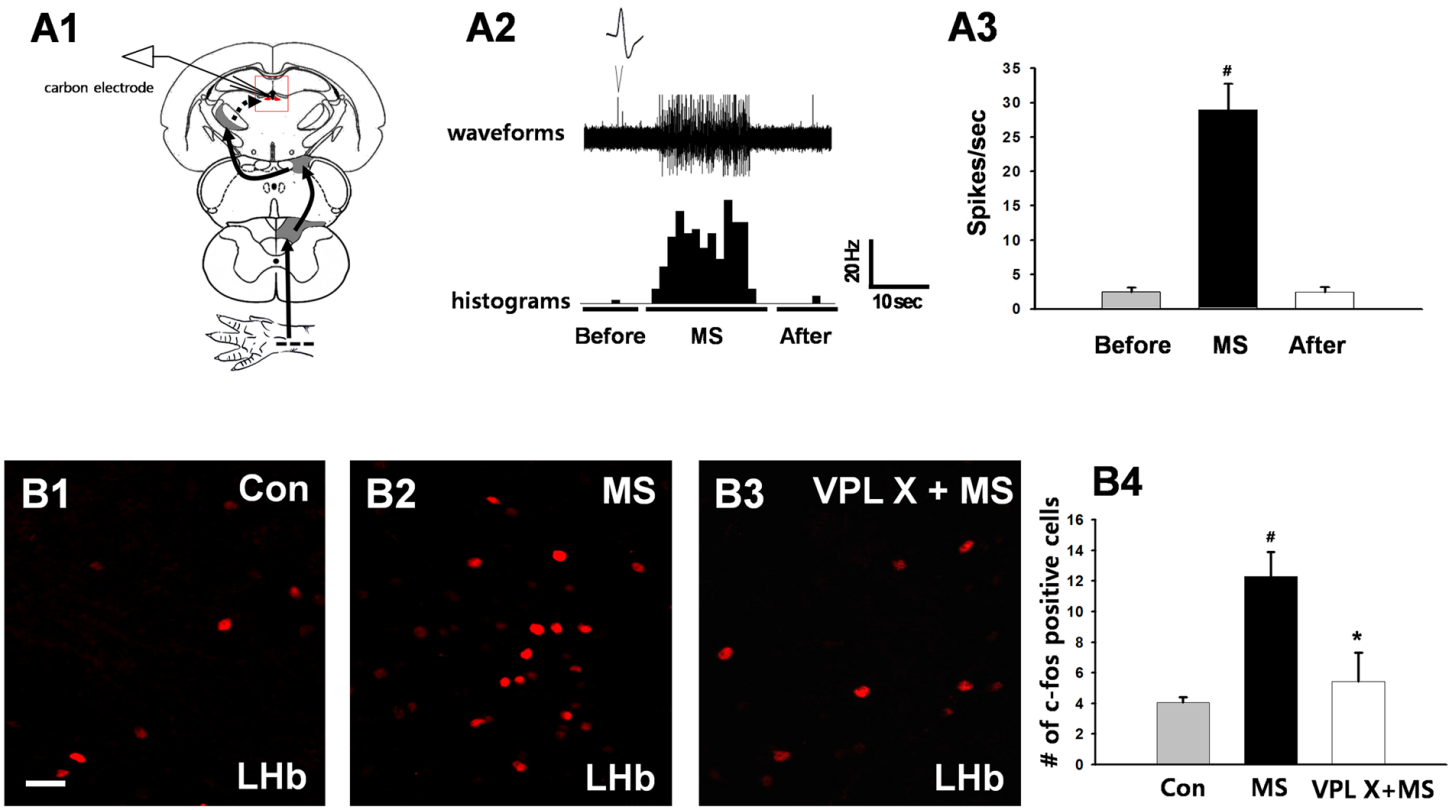
The role of the VPL in the neural activation of LHb following ulnar MS. (**A**) Extracellular single-unit recordings in the LHb during ulnar MS. Representative waveforms (A1) and peri-stimulus time histograms (A2). Single-unit activities of LHb neurons were markedly evoked during ulnar MS (*n* = 7) (A2–3). ^#^ *p* < 0.05 vs. Before. (**B**) Ulnar stimulation (MS, n = 5; B2, B4) significantly increased the numbers of c-fos immunopositive cells, compared to control (Con, *n* = 5; B1, B4), which was prevented in the rats injected with ibotenic acid into VPL (VPL X, *n* = 5; B3–4). ^#^ *p* < 0.05, Con vs. MS; *p < 0.05, MS vs. VPL X + MS. Bar = 20 µm.

The LHb conveys inhibitory reward signals that inhibit VTA DA neurons and DA release in the NAc^19^, and neurons in the LHb can be excited by peripheral stimulation^20^. To determine if ulnar MS might activate the LHb, we measured brain temperature, an indicator of metabolic neural activation^21^. LHb temperature following ulnar MS increased to 0.073 ± 0.013 °C over baseline and recovered to baseline shortly after stimulation (LHb; Fig. 4A1–3), while no significant changes of temperature were seen during ulnar MS in control sites, 1.3 mm apart from LHb (Con; Fig. 4A1–3). To provide evidence for LHb activation and specific LHb projections to the VTA/RMTg, we examined the expression of c-fos in LHb neurons retrogradely labeled from the VTA/RMTg. The number of c-fos positive cells in the LHb increased approximately 2-fold in rats undergoing ulnar MS compared to naïve rats (*p* = 0.003; Fig. 4B1–3). Numbers of c-fos positive cells in retrogradely labeled LHb neurons significantly increased in ulnar MS-treated rats compared to naïve rats (Fig. 4C1–4; Con vs. MS. *P* < 0.05), suggesting that VTA/RMTg-projecting LHb neurons were activated by ulnar MS. To identify the role of the LHb in sensory inhibition of cocaine locomotion, electrolytic lesions of LHb were made bilaterally (Fig. 4D1–2). LHb lesions impaired the inhibition of cocaine locomotion by ulnar MS (LHb X + MS), compared to control group (Sham + MS; Fig. 4D3). To further confirm that the neurons in the LHb were evoked during ulnar MS, extracellular recordings were performed in LHb. The mean firing rates of LHb neurons (n = 21 from 7 rats) during ulnar MS increased from 2.44 ± 0.6 spikes/sec to 28.95 ± 3.8 Hz and returned to baseline (2.43 ± 0.71 Hz) shortly after termination of ulnar MS (*p* < 0.05, Fig. 5A1–3). To see a putative VPL-LHb link, we explored whether VPL lesion could interrupt the neuronal activation of LHb following peripheral stimulation. The c-fos expression in the LHb increased following ulnar MS (12.29 ± 1.62), compared to controls (Con, 4.04 ± 0.27), which was prevented by VPL lesion (VPL X + MS, 5.42 ± 1.88; Fig. 5B4, *p* < 0.05).

### Dorsal Column (DC) Pathways and Lateral Habenula (LHb) Mediate Inhibitory Effects of Ulnar Nerve Stimulation on Cocaine Induced Accumbal activity

**Figure 6:**
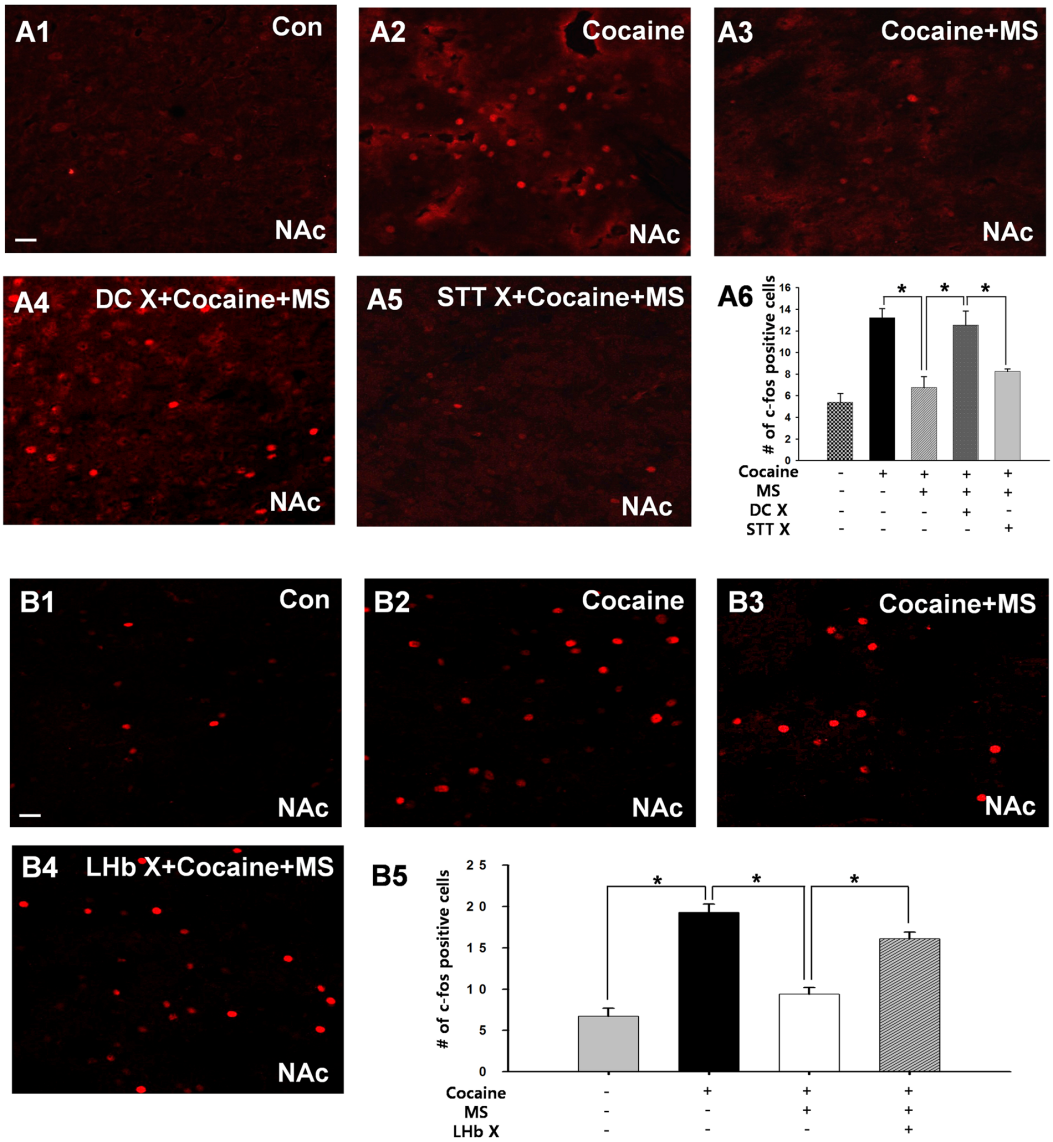
The effects of DC or LHb lesions on ulnar inhibition of cocaine-induced c-fos expression in the NAc. (**A**) The cocaine-induced increase in c-fos positive neurons in NAc (A2; Cocaine, *n* = 6) was attenuated by ulnar stimulation (A3; Cocaine + MS, *n* = 6), which was prevented when the DC was damaged (A4; DC X + Cocaine + MS, *n* = 6), but not STT (A5–6; STT + Cocaine + MS, *n* = 6). A1, control (Con); *p < 0.05, Bar = 20 µm. (**B**) The cocaine-induced increase in c-fos positive neurons in NAc (B2; Cocaine, *n* = 6) was attenuated by ulnar stimulation (B3; Cocaine + MS, *n* = 6), which was prevented when the LHb was damaged (B4–5; LHb X + Cocaine + MS, *n* = 6). B1, control (Con); *p < 0.05, Bar = 20 µm.

To evaluate if ulnar stimulation could suppress NAc activity, we examined the expression of c-fos in the NAc by acute cocaine injection following surgical lesion of either DC, STT or LHb. The numbers of c-fos positive cells were significantly increased in cocaine-treated rats compared to controls. The cocaine-induced increase in c-fos positive neurons was attenuated by ulnar MS, which was prevented when the DC was lesioned, but not when the STT was lesioned (Fig. 6A1–6). In another set of animals, we repeated this experiment after disruption of the LHb. Electrolytic lesion of LHb prior to ulnar stimulation blocked the inhibitory effect of ulnar stimulation on cocaine-induced c-fos expression in the NAc (Fig. 6B1–5).

## Discussion

The DC somatosensory pathway is involved in signaling innocuous information from A-fiber mechanoreceptors that mediate tactile discrimination, well-localized touch, vibration and proprioception^14^. We and others have provided indirect evidence implicating the DC pathway in the modulation of the midbrain DA system. Stimulation of HT7 acupoint over the ulnar nerve (known as Guyon’s Canal) inhibits cocaine-induced locomotion via activation of A-fiber and also prevents DA release and drug-seeking behaviors by ethanol, cocaine and morphine^11–13, 22, 23^. Sciatic nerve stimulation at a low threshold current inhibits the activity of most DA neurons in the substantia nigra^24^. The present study showed that the DC pathway was directly involved in peripheral inhibition of cocaine-induced psychomotor response and suggests a novel functional role of the DC pathway in conveying inhibitory signals from the periphery to the brain reward center. Bilateral stimulation of the forearm radial site did not affect cocaine-induced behaviors, which is consistent with previous studies^11, 25^. Indeed, it was reported that unilateral stimulation of the radial nerve at low thresholds produces opposite effects on DA activity in anesthetized cats, characterized by DA decrease in the contralateral midbrain and DA increase in the ipsilateral midbrain^26^. When bilaterally stimulated as in the present study, the radial nerve did not have a significant effect on midbrain DA activity or cocaine-induced psychomotor behaviors.

Our previous and present studies demonstrated that innocuous, mechanical stimulation of the ulnar nerve at HT7, an acupoint at the ulnar nerve, suppresses drug-seeking behaviors and drug-induced DA release in NAc^11, 12, 25, 27^. During such stimulation, the superficial or deep afferents of ulnar nerve are activated and the afferent signals are transmitted via A-fiber of ulnar nerve^11^. The afferent inputs reduce drug-induced DA release in the NAc by normalizing the decreased activity of VTA GABA neurons in addicted rats^9, 12, 22^. While our previous and present studies demonstrated that under drug-induced conditions, innocuous sensory inputs produced a consistent suppression of psychomotor behaviors and DA release^11, 12, 25, 27^, others have shown mixed results in responses of midbrain DA neurons to innocuous stimulation in drug-naïve conditions. For examples, non-noxious stimuli such as foot pressure and cervical probing elicit short latency facilitation or inhibition of midbrain DA neurons in drug-naïve rats^28^. Innocuous sensory stimulation by electrical stimulation or 80–100 mmHg pressure to the skin can produce a slight decrease in DA release in the dorsal striatum^4, 5^ or a mild increase in DA release in the NAc^15^. This discrepancy may be due to anatomical segregation of functional subgroups in midbrain DA neurons responding to sensory stimuli. In support of this, one previous study demonstrated that DA neurons activated by sensory stimuli are located dorsolaterally in the substantia nigra pars, while DA neurons inhibited by the stimuli are found ventromedially, some in the VTA^29^. In the present study, the afferent inputs, which were conveyed via DC pathway, attenuated cocaine-induced c-fos expression in NAc. It led to the speculation that afferent inputs from cutaneous innocuous stimuli would inhibit DA neurons in the VTA causing a reduced DA release in NAc, thereby suppressing cocaine-induced psychomotor behaviors.

How does the activation of DC pathways by ulnar MS suppress drug-seeking behaviors? The LHb, an epithalamic structure, projects to the VTA and the RMTg, which contain GABA neurons that inhibit DAergic activity and the behavioral effects of drugs of abuse^19^. Electrical stimulation of LHb neurons elicits a strong inhibition of VTA DA neurons, while inhibition of the LHb is associated with the activation of DA neurons^18^. Activation of the habenula reduces addiction to cocaine, nicotine, morphine and ethanol^19, 30, 31^, while lesions of the habenula increase drug seeking behaviors^32^. Our findings demonstrate that ulnar MS increased both local temperature and c-fos expression in LHb neurons projecting to the VTA/RMTg regions and LHb lesions blocked ulnar inhibition of cocaine locomotion, indicating that excitation of habenula neurons by ulnar MS can suppress psychomotor responses or NAc-neuronal activity to cocaine (Figs 4–6). Taken together, these findings provide convincing evidence that the DC somatosensory pathway is functionally linked to the LHb-VTA/RMTg pathway, which may underlie somatosensory inhibition of cocaine-induced psychomotor behaviors. Although the LHb has been shown to receive somatosensory inputs^16, 20^, it is not known yet how somatosensory inputs enter the LHb, which receives input from structures, such as the prefrontal cortex, lateral hypothalamus and entopeduncular nucleus^33^. As there are no known direct pathways projecting from VPL to the LHb, linking DC somatosensory inputs to the LHb may require multi-synaptic pathways. Lesions of the VPL abolished neuronal activation of LHb by peripheral stimulation, suggesting a putative VPL-LHb link. Others have shown that the VPL projects to prefrontal cortex^34^, which sends excitatory inputs to the LHb^35^. Connections between the VPL and LHb may be linked via the prefrontal cortex, which requires further elucidation. Regardless, previous studies have demonstrated that LHb neurons are sensitive to noxious stimuli. For example, LHb neurons respond to peripheral noxious stimuli^16^ and the expression of the immediately early gene c-fos in LHb is induced after noxious stimuli^36^. Our findings suggest that innocuous stimulation of the ulnar nerve would activate LHb neurons, as measured by local temperature, c-fos expression and *in vivo* extracellular recordings in the LHb. Thus, there is the possibility that noxious as well as innocuous stimuli can activate LHb neurons under certain conditions and inhibit psychomotor response to cocaine.

The present study revealed that stimulation of the ulnar nerve suppressed cocaine-induced hyperlocomotion and the effects were completely abolished by lesions to the DC pathway and LHb, but not to the STT. Additionally, activation of the DC pathway or the LHb was detected following ulnar MS. These findings strongly suggest that the DC somatosensory pathway conveys an inhibitory signal from the periphery to mesolimbic reward circuits and the activation of spinal DC pathway can inhibit psychomotor response to cocaine.

## Methods

### Animals and Ethics Statement

Male Sprague-Dawley rats (270–320 g, Daehan Animal, Seoul, Korea) were used for this study. All rats had free access to food and water and were maintained on a 12 hr light-dark cycle. All procedures were approved by the Institutional Animal Care and Use Committee at the Daegu Haany University and conducted in accordance with National Institutes of Health guidelines for the care and use of laboratory animals. Each group consisted of 6–8 rats, unless stated otherwise.

### Chemicals

Cocaine (15 mg/ml saline; Macfarlan Smith Ltd., UK), Fluorogold (20 mg/ml saline; a retrograde tracer; FG; Fluorochrome, USA) and ibotenic acid (5 mg/ml saline; Sigma, USA) were used.

### Cocaine-induced Locomotor Activity

Locomotor activity was measured with an image analysis system (Ethovision 3.1, Noldus Information Technology BV, Netherlands) as previously described^11^. Briefly, each animal was placed into a square open field box (40 cm × 40 cm × 45 cm) and monitored with an overhead video camera and video tracking software. After recording baseline activity for 30 min, the animal was given an intraperitoneal (i.p.) injection of cocaine (15 mg/kg) and monitored for up to 60 min after injection. The distance travelled during each 10-min period was analyzed. The data are expressed as a percentage of baseline activity.

### Mechanical Stimulation (MS)

Since our previous studies showed that mechanical stimulation of a needle inserted into the ulnar tunnel (Guyon’s Canal or HT7 acupuncture *Shenmen Point*) in the forelimb produces intense and reproducible inhibitory effects on various addictive behaviors caused by cocaine, morphine and ethanol^9, 11, 12, 22^, a peripheral stimulation was performed as described in our previous experiments^11^. Mechanical stimulation was performed about 1 min after cocaine injection. Briefly, while an assistant lightly restrained the rat, needles (0.10 mm in diameter, 10 mm in length of needle; Dongbang Medical Co., Korea) were inserted bilaterally 3 mm deep into the ulnar tunnel on the transverse crease of the wrist of the forepaw. By using a newly-developed mechanical instrument that consisted of a custom-made control unit and a mechanical vibrator connected to the needle, the inserted needle was mechanically stimulated for 20 sec in duration at an intensity of 1.3 m/sec^2^, maintained for 1 min after needle insertion and subsequently withdrawn. Following mechanical stimulation, locomotor activity was monitored for 60 min after cocaine injection. To assess the possibility that the mechanical stimulation of the wrist impaired locomotor behaviors, the radial side of wrist joint was mechanically stimulated in an identical fashion as a control condition.

### Surgical Transection of Ulnar Nerves

A small skin incision was made longitudinally on the medial part of the elbow to expose the ulnar nerve under isoflurane anesthesia. The ulnar nerve was bilaterally ligated with 4–0 silk and cut around the medial head of the triceps muscle of both forelimbs. Forty-eight to 72 hr after the nerve injury, the rats were subjected to the locomotor activity test following acute cocaine injection.

### Surgical Lesions of the Dorsal Column (DC) or Spinothalamic Tract (STT) Pathways

Lesions of DC or STT were performed as previously described^37, 38^ with slight modifications. Briefly: (1) dorsal column lesions were made bilaterally to a depth of 0.4 mm into the cord using fine tipped Dumont #5 forceps (Fine Science Tools, USA) with aid of a dissecting microscope after a laminectomy at the C3 portion of the spinal cord; (2) For surgical lesions of cuneate nucleus (CN; second-order neurons of DC pathway), the animal was placed in the stereotaxic frame with the head tilted forward, the obex was exposed surgically and the cuneate nucleus was macerated with the fine forceps along its length; (3) For lesioning the STT pathway, the ventrolateral funiculus (VLF) at the C3 portion of the spinal cord was lifted with a fire polished glass rod and the tip of a 22 gauge needle was used to damage the left and right ventrolateral quadrants. Animals in the sham group in each experiment were subjected to a laminectomy or surgical exposure of the obex without damaging the nerve. The rats were allowed to recover for at least 7 days after surgery.

### Lesions of Ventral Posterior Lateral (VPL) Thalamus and Lateral Habenula (LHb)

For VPL lesions, ibotenic acid (0.5 μl/site) was bilaterally microinjected (third-order neurons of the DC pathway)^39^. The anesthetized rat (pentobarbital, 50 mg/kg, i.p.) was placed in a stereotaxic frame, and two holes were drilled in the skull to access the following coordinates: anterior, −2.5~−3 mm; lateral, ±2.75~3 mm; deep, 6 mm^40^. A 26-gauge Hamilton syringe (Reno, NV, USA) filled with either ibotenic acid or saline was infused at a rate of 0.5 μl/min using a microinjection pump (Pump 22, Harvard Apparatus, USA). The syringe was left in place for at least 5 min to facilitate diffusion after injection. For electrolytic lesion of bilateral LHb, tungsten electrodes insulated except at 0.5 mm tip were inserted in the following coordinates: anterior, −3.5 mm; lateral, ±0.7 mm, deep −4.9 mm. Lesions were made by passing ± 0.35 mA of DC current for 8 sec alternately. The rats were allowed to recover for at least 7 days after surgery.

### Histological Examination of Lesions

At the termination of the experiments, all rats were sacrificed for histological confirmation of lesions. All lesions were confirmed by toluidine blue stain. The animals were perfused with phosphate-buffered saline (PBS) and then with 4% paraformaldehyde. The brains and spinal cords were removed, post-fixed in 4% paraformaldehyde and cryoprotected in 30% sucrose. The tissue was then sectioned into 30 μm-thick sections using a cryostat (Leica CM 1850; Leica Biosystems, Germany) and stained with toluidine blue. Only those rats with histologically-verified lesions were included in the data analysis.

### Extracellular Recording in the Cuneate Nucleus

The *in vivo* extracellular single unit recordings in the CN were performed according to a previously published method^41^. In brief, rats were deeply anesthetized with an intraperitoneal (i.p.) injection of sodium pentobarbital (60 mg/kg) for surgery, were mounted on the stereotaxic frame, and were supplemented with sodium pentobarbital (5 mg/kg/h) infused intravenously through a jugular vein catheter during the recording process to maintain anesthesia. The adequacy of anesthesia was monitored by the lack of withdrawal reflexes to noxious stimuli and the absence of corneal blink reflexes. Body temperature was maintained at 37 °C with a thermostatically controlled heating blanket with a rectal probe. Extracellular potentials of single neurons in the CN were recorded using a carbon-filament glass microelectrode (Carbostar-1, Kation Scientific, USA) at a depth of 0.0–0.8 mm ventral from the dorsal surface of the medulla, 1 mm caudal to the obex and 1–2 mm lateral from the midline^40^. Neurons were classified as low-threshold (LT) or wide-dynamic range (WDR) neurons based on the response to mechanical somatic stimuli. LT neurons were excited by only innocuous stimuli (light pressure), and WDR neurons responded to both innocuous (light pressure) and noxious stimuli (blunt clip). The signals were amplified (Iso80, World Precision Instruments, USA) and recorded using a micro1401 and Spike2 software (Cambridge Electronic Design, UK).

### Extracellular Recording of Lateral Habenula (LHb) Neurons

Single-unit electrophysiological recordings in the LHb were performed with a slight modification as described previously^42^. In brief, rats (n = 7) were placed in a stereotaxic frame under pentobarbital anesthesia (60 mg/kg, i.p.). Anesthesia level was maintained by intravenous infusion of sodium pentobarbital (15 mg/kg/hr) during the recording. A single microcarbon (7 μm diameter) filament-filled glass electrode (carbon fiber electrode, impedence 0.4–1.2 MΩ, Kation Scientific, USA) was positioned into the LHb (anterior, −3.5 mm; lateral ± 0.7 mm; deep, −4.9 mm) with one-axis water hydraulic micromanipulator (Narishige, USA). Neuronal signals were amplified 10,000 times, filtered at 0.3 to 10 kHz and digitized by using an Iso80 amplifier and a microCED-1401. Single-unit activity was isolated by Spike2 software. Once stable baseline activity was achieved for at least 10 min, mechanical stimulation was applied to the ulnar site for 20 sec. Firing rates were evaluated for 20 sec before, 20 sec during and 20 sec after mechanical stimulation.

### Immunohistochemistry for c-fos Expression

Brain or spinal cord sections were removed after perfusion with 4% paraformaldehyde, postfixed and cryosectioned into 30 µm-thick sections at the level of the CN (coordinates: anterior, −14.30~−14.60 mm; lateral, ±1.0~1.4 mm; deep, −7.8~−8.2 mm), the NAc (coordinates: anterior, 1.60~1.30 mm; lateral, ±0.6~1.0 mm; deep, −6.8~−7.4 mm) or the LHb (coordinates: anterior, −3.5 mm; lateral, ±0.7 mm; deep, −4.9 mm). The sections were incubated overnight (approximately 16 hr) at 4 °C with anti-c-fos rabbit polyclonal antibodies (1:200; Sigma, USA) followed by a 2-hr incubation at room temperature with a biotinylated donkey anti-rabbit Alexa Fluor 594 (red; 1:500; Sigma, USA) and mounted onto gelatin-coated slides. The sections were photographed and quantified using confocal laser scanning microscopy (LSM700, Carl Zeiss, Germany).

### Retrograde Labeling of Habenular Neurons Projecting to the Ventral Tegmental Area (VTA)/Rostromedial tegmental Nucleus (RMTg)

After placing the animal into a stereotaxic frame under pentobarbital anesthesia (50 mg/kg, i.p.), the retrograde tracer Fluorogold (0.1 µl) was injected bilaterally to diffuse into the VTA/RMTg region (coordinates: anterior, −6.7 mm; lateral, 0.6 mm; deep, 8 mm) using a 5 μl Hamilton syringe controlled by a syringe pump at a rate of 0.1 μl/3 min. At least 4 days were allowed for the dye to diffuse.

### Measurement of Habenular Temperature

The local brain temperature is monitored as an indicator of functional neural activation^21^. Temperature changes in the LHb following peripheral stimulation were monitored as described previously^43^ with slight modifications. In brief, under pentobarbital-induced anesthesia (50 mg/kg, i.p.), an access hole was drilled through the skull over the habenula (coordinates: anterior, −3.5 mm; lateral, ±0.7 mm; deep, −4.9 mm) or the control site (coordinates: anterior, −3.5 mm; lateral, ±2.0 mm; deep, −4.9 mm), and a thermocouple needle microprobe (NJ-07013, World Precision Instruments, USA) was slowly lowered to the desired target depth. The thermocouple probe was connected to a BAT-12 digital thermometer (Physitemp, USA) and a data acquisition system (Powerlab, ADinstrument, Australia). The LHb temperature was measured while body temperature was maintained at approximately 36.5 °C with a heating pad.

### Data Analysis

All data are presented as the means ± standard error of the mean (SEM) and analyzed by one- or two-way repeated measurement analysis of variance (ANOVA) followed by post hoc testing using the Tukey method or *t*-test, where appropriate. Statistical significance was considered at *p* < 0.05.
